# Glycogen depletion can increase the specificity of mucin detection in airway tissues

**DOI:** 10.1186/s13104-018-3855-y

**Published:** 2018-10-25

**Authors:** David K. Meyerholz, Amanda P. Beck, J. Adam Goeken, Mariah R. Leidinger, Georgina K. Ofori-Amanfo, Hannah C. Brown, Thomas R. Businga, David A. Stoltz, Leah R. Reznikov, Heather A. Flaherty

**Affiliations:** 1 Department of Pathology, 1165ML, University of Iowa Carver College of Medicine, Iowa City, IA 52242 USA; 2 Department of Pathology, Albert Einstein College of Medicine, Bronx, NY USA; 3 Department of Internal Medicine, University of Iowa Carver College of Medicine, Iowa City, IA USA; 4 Department of Molecular Physiology and Biophysics, University of Iowa Carver College of Medicine, Iowa City, IA USA; 5 Department of Physiological Sciences, College of Veterinary Medicine, University of Florida, Gainesville, FL USA; 6 Department of Veterinary Pathology, Iowa State University College of Veterinary Medicine, Ames, IA USA

**Keywords:** Mucus, Mucin, Periodic acid Schiff (PAS), Diastase-periodic acid Schiff (dPAS), Alcian blue, Glycogen, Staining, Specificity, Scoring, Nonspecific staining

## Abstract

**Objective:**

Mucin is an important parameter for detection and assessment in studies of airway disease including asthma and cystic fibrosis. Histochemical techniques are often used to evaluate mucin in tissues sections. Periodic acid Schiff (PAS) is a common technique to detect neutral mucins in tissue, but this technique also detects other tissue components including cellular glycogen. We tested whether depletion of glycogen, a common cellular constituent, could impact the detection of mucin in the surface epithelium of the trachea.

**Results:**

Normal tissues stained by PAS had significantly more staining than serial sections of glycogen-depleted tissue with PAS staining (i.e. dPAS technique) based on both quantitative analysis and semiquantitative scores. Most of the excess stain by the PAS technique was detected in ciliated cells adjacent to goblet cells. We also compared normal tissues using the Alcian blue technique, which does not have reported glycogen staining, with the dPAS technique. These groups had similar amounts of staining consistent with a high degree of mucin specificity. Our results suggest that when using PAS techniques to stain airways, the dPAS approach is preferred as it enhances the specificity for airway mucin.

## Introduction

Pathological evaluation of tissues is a cornerstone approach for assessment in biomedical studies, especially those studying animal models of human disease [1]. Histochemical stains are frequently used tools to assist in the examination of tissues. For instance, hematoxylin and eosin (HE) is a technique that produces a characteristic appearance in tissues for routine examination. In contrast, other histochemical stains can bind to specific biological substances and serve as markers used in scoring tissue changes of disease conditions.

In the lung, mucus is an important tissue parameter in the study of several airway diseases including asthma [2–4], and cystic fibrosis [5, 6]. Some of the more common histochemical techniques that have been used for mucus detection in tissues include alcian blue (AB) and periodic acid Schiff (PAS). AB is reportedly specific for acidic mucins and PAS is reportedly specific for neutral mucins [7–10]. One potential limitation to histochemistry is the fact that many stains have limited specificity, meaning they can sometimes also stain other tissue parameters. For instance, the PAS technique is also commonly applied to tissues for detection of glycogen [11, 12], basement membranes [13, 14] and certain fungal pathogens [15]. Basement membranes and pathogens can often be distinguished readily by their morphology; however, glycogen is a cytoplasmic constituent of cells and its morphologic distinction versus cellular mucins can at times be challenging. In this study, we evaluated whether glycogen stores in normal airway surface epithelial could influence the detection of mucins. We found that glycogen significantly increased the PAS staining of airway tissues compared to diastase-treated tissues with PAS stain (dPAS). The extent of airway epithelial staining using dPAS and AB techniques were similar indicating a high specificity for cellular mucins. These findings suggest that glycogen significantly confounds PAS staining in airways and that use of the dPAS technique can increase the specificity for mucin to avoid false positive interpretations.

## Main text

### Methods

Mouse livers and pig tracheas were secured from a de-identified tissue repository and these blocks were used as control tissues in various histochemical and immunohistochemical stains. For pig tracheas (n = 8, both sexes, < 1 week of age), each tissue block had two sets of serial sections (~ 4 µm) made for comparison of stains. One set (group comparison 1) for comparison of dPAS versus PAS techniques and one set (group comparison 2) for dPAS versus AB techniques.

Histochemical staining was performed using standard PAS and AB techniques [10, 16, 17]. Relevant to this study, it is important to note that PAS and dPAS techniques were identical in nature except that dPAS groups had pre-treatment with diastase (i.e. amylase) enzyme to deplete the glycogen stores in the serial sections of tissue [18]. Briefly to deplete the glycogen, deparaffinized slides were incubated with gentle agitation in a 0.46% solution of commercial enzyme (Sigma A-3176, Sigma Life Science, 37 °C for 20 min). Slides were then washed and ready for the PAS technique.

Serial sections were evaluated for high quality tissue (i.e. lack of tissue artifacts) and digital images were collected with a high resolution digital camera (200× magnification, DP73 on a BX 51 microscope, Olympus). Specialized software (CellSens, Olympus) was used for morphometry and thresholds for digital images were defined for dPAS stained tissue (hue: 256 min, 321 max; saturation: 117 min, 256 max; and intensity value: 48 min, 221 max) to analyze dPAS and PAS samples. AB thresholds were independently defined in an AB stained serial section (hue: 181 min, 212 max; saturation: 98 min, 256 max; and intensity value: 48 min, 256 max) and used exclusively for the AB stained tissues. Tissue staining thresholds were defined by the same pathologist with the goal to maximize specific staining on surface epithelium (SE) goblet cell mucin and minimize non-specific background staining. In each image, the SE was defined as a region of interest (ROI) from the apical surface to the basement membrane and then the % area of mucin staining was evaluated within the ROI. The % results for each tissue’s PAS and AB analyses were enumerated and then normalized for each comparison relative to the respective serial section of dPAS (i.e. PAS [% area]/dPAS [% area], and AB [% area]/dPAS [% area]) to yield a value of “1” for dPAS.

For semiquantitative scoring, we followed published principles to maximize reproducibility of tissue scoring [19, 20]. Pathologists were masked to groups using either the group masking (i.e. pathologist given only treatment group affiliation such as group A, group B, etc.) or post-examination masking (i.e. pathologist able to screen the slides in a fully informed manner to develop scoring approaches and then was masked to treatment groups for scoring) methods. The ability of the stain to detect mucin in goblet cells was scored in the following manner: (A) detection of mucin—“1”, mucin staining in SE goblet cells was detectable, but absent (clear space) or weakly/partially stained in multiple goblet cells; “2”, mucin staining was detectable in goblet cells and distinct with rare goblet cells with weak/absent staining; and “3”, mucin staining was robust with crisp and distinct delineation within most all goblet cells; and B) detection of nonspecific staining in SE (e.g. ciliated cells) was evaluated in the following manner: “1”—absent to rare, “2”—multifocal (1–50% of SE cells), and “3”, extensive (> 50% of SE cells).

Statistical analysis was performed using Prism (v7.04, Graphpad Software) and in a manner that suitably matched the statistical test with the experimental design and data [1]. As the tissues in each group comparison were paired, we use the Wilcoxon matched-pairs signed rank test to evaluate the comparisons. Statistical significance was defined at P < 0.05.

### Results

**Fig. 1:**
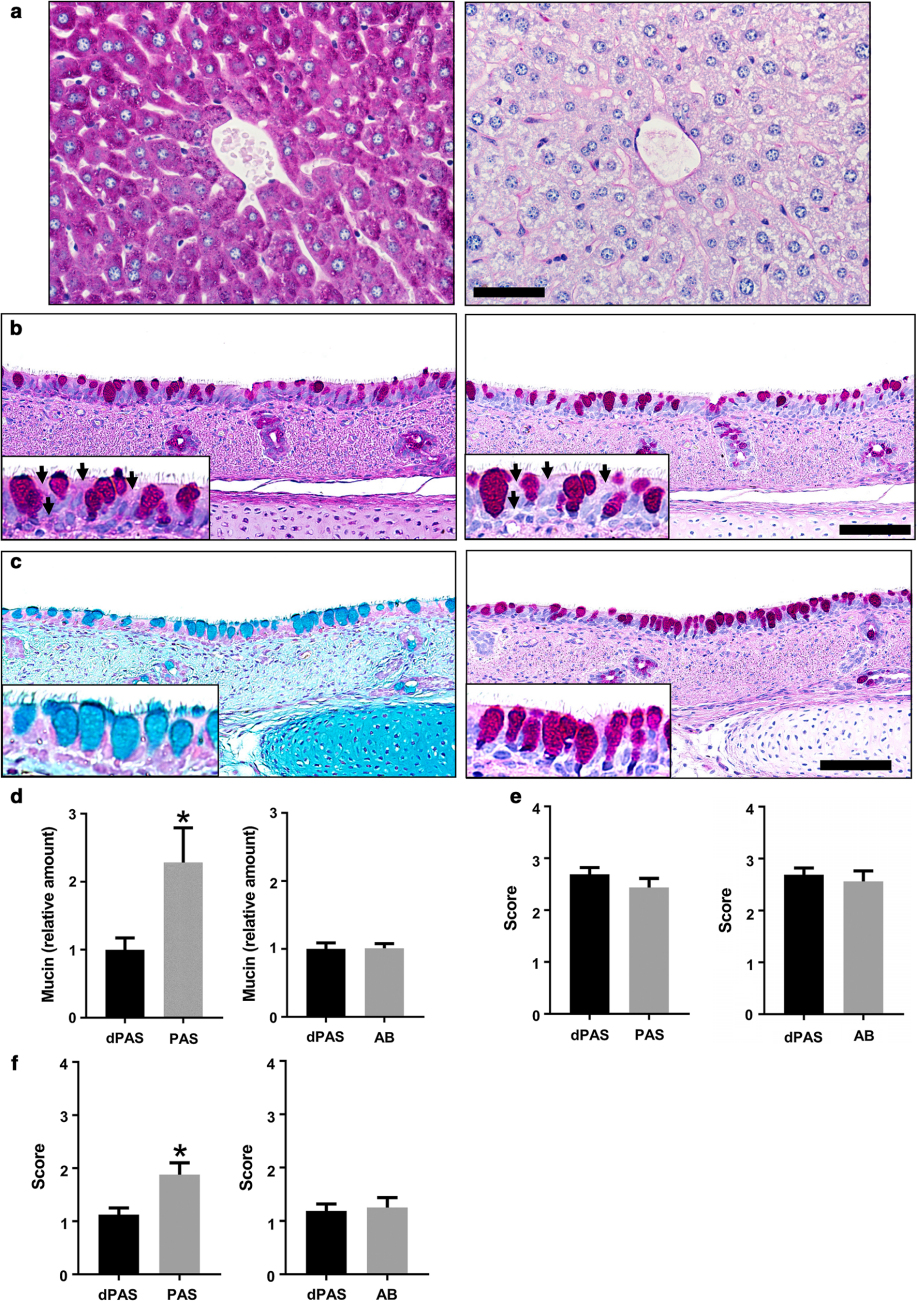
Images and scores from PAS, dPAS and AB stained tissues sections. **a** Mouse liver with PAS (left) and dPAS (right) stains, bar = 48 µm. Note the abundant and widespread cytoplasmic magenta staining in the PAS, but absent in the dPAS livers. **b** Pig trachea with PAS and dPAS stains, bar = 85 µm. Note the ciliated cells (arrows, insets) with cytoplasmic magenta staining in PAS, but absent in PAS trachea. **c** Pig trachea with AB and dPAS stains, bar = 85 µm. Note that AB and dPAS techniques preferentially stain mucin in goblet cells, but lack nonspecific cytoplasmic staining of adjacent ciliated cells as seen in **b**. **d**–**f** Evaluation of PAS, dPAS, and AB mucin staining in the trachea. **d** Extent of mucin in surface epithelium (SE) normalized to serial dPAS sections (value of “1”), bars = mean ± sem. The presence of glycogen significantly increased the PAS versus dPAS staining of SE (P = 0.0078). Comparison of dPAS and AB showed no significant differences (P > 0.9999) even though the tissues still had glycogen for the AB technique. **e** Ordinal scores for mucin staining within SE goblet cells showed no significant differences (P = 0.125 and 0.625, respectively). **f** Ordinal scores for nonspecific mucin staining in ciliated cells of the SE showed increased PAS versus dPAS scores, but no difference in AB and dPAS scores (P = 0.0156 and 0.9999, respectively)

To demonstrate the potential impact of cellular glycogen stores using PAS versus dPAS techniques, we examined serial sections of mouse livers (n = 2). Liver is known to be a storage organ for glycogen and accordingly the PAS section had ample magenta staining, but with dPAS samples (depleted of the glycogen) had a profound loss/absence of magenta staining (Fig. 1a).Because of this overt contrast in glycogen staining, the liver is a commonly used control tissue in pathology laboratories to validate glycogen depletion for dPAS stains.

We evaluated serial sections of trachea to determine the extent SE staining, as goblet cell mucin is commonly targeted for examination in airway SE. The PAS technique appeared to have produced more SE staining compared to the dPAS section, most of the difference in staining was localized in the ciliated cells versus goblet cells (Fig. 1b). Evaluation of AB relative to dPAS techniques showed no overt differences beyond the minor amount expected for serial sections (Fig. 1c). To further validate these findings we quantitatively analyzed digital images and compared serial sections of group comparison 1 (PAS and dPAS) and group comparison 2 (AB and dPAS). In group comparison 1, PAS had significantly greater staining compared to serial dPAS sections (Fig. 1d). The excess staining of the SE cannot be due to mucins, but is rather is indicative of the presence of glycogen in PAS stained tissues. In group comparison 2, AB and dPAS had nearly identical extent of SE staining, consistent with mucin-centric affinity for both techniques. AB does not have a reported affinity for glycogen as does PAS and this study confirms that view.

Pathologists and biomedical personnel who evaluate tissues, both desire stains with good sensitivity (i.e. ability to detect staining at the target of interest) and specificity (i.e. ability to avoid staining at off-target tissues) [21]. To evaluate if a trained observer could detect differences in these staining techniques, we had two pathologists (masked to groups) score the tissues for sensitivity (i.e. ability to detect mucin in SE goblet cells, Fig. 1e) and specificity (i.e. ability to avoid staining non-targeted SE cells, Fig. 1f). The ability of the stains to detect mucin within SE goblet cells was observed to be similar across the stains in both group comparisons (Fig. 1e). However, the pathologists were able to observe significantly more nonspecific staining in PAS versus dPAS techniques, but no significant differences were seen in AB versus dPAS techniques (Fig. 1f). The similar staining was consistent with previous observations that SE goblet cells in cartilaginous airways can produce more than one type of mucin [10, 22].

### Discussion

Histochemical stains are a mainstay of tissue evaluation. The literature has several examples of histochemical stains that are specific for certain tissue parameters, but the specificity of a histochemical stain is more of a relative than exclusive concept in many circumstances. This fact is not easily recognized by the inexperienced observer. For instance, toluidine blue is a stain used to specifically identify mast cells by its metachromatic coloration; however, mucins in goblet cells can also exhibit metachromatic coloration. This can create challenges when quantifying metachromatic mast cells versus metachromatic goblet cells in airway epithelium [23, 24]. Similarly, PAS is often defined as being specific for neutral mucins and, by default, PAS staining of the airway SE can be hastily interpreted as being mucin without consideration for glycogen. PAS by itself, or in combinatorial stains such as AB/PAS [25], have been used to detect airway mucins, but without glycogen depletion (as with the dPAS technique) these approaches could be prone to false positive staining, because of the remnant glycogen. Use of the dPAS technique in airway evaluation is performed by some investigators [2, 17, 26], but the objective need for using this technique in airway tissues has not been clearly demonstrated until now. Our results suggest that glycogen can have a significant impact in airway tissue evaluation and the dPAS technique should be preferentially used to maximize the mucin specificity of the stain.

### Limitations

This study is not without potential limitations. First, we have utilized a relatively small number of samples (n = 8), but we used paired tissue samples to strengthen the confidence in the results. Second, the quantitative analyses of SE mucin relied on specific thresholds defined by a pathologist and it is possible that using variations in threshold settings could influence the final extent of changes. Lastly, the amount of glycogen stores impacting our airway tissues may not fully replicate that of glycogen stores in other ages of pigs or even other species. The fact that the pig has proven to be a successful model species for the study of airway pathophysiology [5, 6, 27, 28] could suggest that these results are broadly useful.

## Abbreviations

AB: alcian blue
dPAS: diastase-periodic acid Schiff
HE: hematoxylin and eosin
PAS: periodic acid Schiff
ROI: region of interest
SE: surface epithelium
